# Hedgehog signaling regulates hypoxia induced epithelial to mesenchymal transition and invasion in pancreatic cancer cells via a ligand-independent manner

**DOI:** 10.1186/1476-4598-12-66

**Published:** 2013-06-20

**Authors:** Jianjun Lei, Jiguang Ma, Qingyong Ma, Xuqi Li, Han Liu, Qinhong Xu, Wanxing Duan, Qing Sun, Jun Xu, Zheng Wu, Erxi Wu

**Affiliations:** 1 Department of Hepatobiliary Surgery, First Affiliated Hospital of Medical College, Xi’an Jiaotong University, 277 West Yanta Road, Xi’an 710061Shaanxi Province, China; 2 Department of Oncology, First Affiliated Hospital of Medical College, Xi’an Jiaotong University, 277 West Yanta Road, Xi’an, 710061Shaanxi Province, China; 3 Department of Pharmaceutical Sciences, North Dakota State University, Sudro Hall 203, Fargo, ND, 58105USA

**Keywords:** Hedgehog signaling, Epithelial to mesenchymal transition, Hypoxia, Invasion, Pancreatic cancer

## Abstract

**Background:**

Hypoxia plays a vital role in cancer epithelial to mesenchymal transition (EMT) and invasion. However, it is not quite clear how hypoxia may contribute to these events. Here we investigate the role of Hedgehog (Hh) signaling in hypoxia induced pancreatic cancer EMT and invasion.

**Methods:**

Pancreatic cancer cells were cultured under controlled hypoxia conditions (3% O2) or normoxic conditions. HIF-1α siRNA, cyclopamine (a SMO antagonist) and GLI1 siRNA were used to inhibit HIF-1α transcription or Hh signaling activation. The effect of hypoxia and Hh signaling on cancer cell EMT and invasion were evaluated by Quantitative real-time PCR analysis, Western blot analysis and invasion assay.

**Results:**

Here, we show that non-canonical Hh signaling is required as an important role to switch on hypoxia-induced EMT and invasion in pancreatic cancer cells. Moreover, our data demonstrate hypoxia induces EMT process as well as invasion, and activates the non-canonical Hh pathway without affecting sonic hedgehog homolog (SHH) expression. Moreover, these effects are reversible upon HIF-1α siRNA interference with unchanged SHH and patched1 (PTCH1) level. Furthermore, our data demonstrate that hypoxia induced invasion and EMT process are effectively inhibited by Smoothened (SMO) antagonist cyclopamine and GLI1 siRNA. In addition, GLI1 interference inhibited EMT progress with significantly suppressed vimentin expression, whereas inhibition of SMO through cyclopamine could not reduce vimentin level. This data indicate that hypoxia could trigger other factors (such as TGF-β, KRAS or RTK) bypassing SMO to activate GLI1 directly.

**Conclusions:**

Our findings suggest that Hh signaling modulates hypoxia induced pancreatic cancer EMT and invasion in a ligand-independent manner. Thus, Hh signaling may represent a promising therapeutic target for preventing pancreatic cancer progression.

## Background

Accompanying with a 5-year survival rate less than 5% and more than 37, 000 deaths per year, pancreatic ductal adenocarcinoma represents one of the most lethal human cancers and is the fourth leading cause of cancer-related deaths in the United States [1,2]. Its high tendency to metastasize is considered to partially account for the extremely poor clinical prognosis of pancreatic cancer [3]. However, the underlying molecular mechanisms of the invasion and metastasis of pancreatic cancer remain poorly understood.

Epithelial to mesenchymal transition (EMT) is a process defining the progression that cells lose their polarized epithelial character and acquire a migratory mesenchymal phenotype [4]. EMT plays a pivotal role in normal physiological development and enables the cancer cells to gain migratory and invasive properties consequently lead to tumor metastasis [5]. An important hallmark of EMT is the loss of the homophilic cell adhesion molecule E-cadherin, which is considered as a main determinant of epithelial cell-cell adhesion and cell polarity [6]. This crucial event has found to be resulted from transcriptional repression of E-cadherin through overexpression of several different EMT-inducing factors, such as Snail, a zinc-finger transcription repressor [7].

Solid tumors often experience low oxygen tension environments, which is predominantly caused by abnormal vasculature formation of the rapidly growing tumor mass. Tumor hypoxia is associated with enhanced tumor invasiveness, angiogenesis, and distant metastasis [8-10]. The adaptation of tumor cells to hypoxia leads to tumor heterogeneity and the selection of resistant clones, consequently evolving into a more malignant phenotype [11]. A transcription factor hypoxia inducible factor-1α (HIF-1α), which mediates hypoxia responses, is overexpressed in many solid tumors, including pancreatic cancer [11]. Stabilization and activation of HIF-1α/HIF-1β transcription complex trigger its target genes related to cell proliferation and metastasis, which correlates with many different cellular processes, such as proliferation, angiogenesis, and EMT [12-15], and poor prognosis and tumor metastasis in cancer patients [13,16,17]. HIF-1α consists of a bHLH domain close to the amino (N) terminal, which is required for DNA binding to hypoxia-response elements to activate the HIF target genes such as endothelin-1, vascular endothelial growth factor (VEGF), and erythropoietin [18].

The Hedgehog (Hh) signaling pathway, which is normally quiescent in adult pancreas, has been shown to be very active in pancreatic cancer where it promotes stromal hyperplasia, myofibroblast differentiation, and production of extracellular matrix (ECM) [19,20], which may promote cancer cells to undergo EMT process to further facilitate the strong propensity of pancreatic cancer for invasion and metastasis. Without binding to Hh ligands, patched1 (PTCH1) holds Smoothened (SMO), a seven transmembrane spanning protein, in an inactive state and thus prohibits signaling to downstream genes. Upon binding to Hh ligands, SMO dissociates from PTCH1 and the signaling is transduced, leading to the activation of target genes, including PTCH1, by transcription factor GLI1 [21-24]. Therefore, expression of SMO and GLI1 is presumed to be the markers of the Hh pathway activation. Another study demonstrates that Hh signaling activation is a very common event in pancreatic cancer, evidenced by the expression of PTCH1 and GLI1 in seven available pancreatic cancer cell lines and 54 pancreatic cancer surgical specimens [25]. In pancreatic cancer, the activation of the Hh pathway could induce an EMT, which leads to invasion and metastasis through down-regulating E-cadherin expression and up-regulating vimentin expression [26,27]. Moreover, a number of signal transduction pathways, including Hh signaling, could be activated in human pulmonary arterial smooth muscle cells under hypoxia conditions [28] or in ischemia tissues [29].

In this study, we focused on elucidating the regulation of EMT and invasion processes in hypoxia condition via Hh signaling, in a panel of pancreatic cancer cell lines. We found that non-canonical Hh signaling in pancreatic cancer cells is a critical mechanism for hypoxia in regulating the process of EMT and invasion.

## Results

### GLI1 and HIF-1α are expressed in pancreatic cancer cell lines

**Figure 1:**
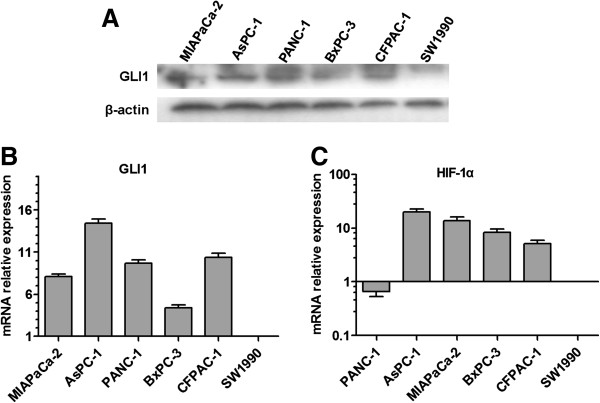
**The expression of GLI1 and HIF-1α in human pancreatic cancer cell lines.** (**A**) The expression of GLI1 at protein level in MIAPaCa-2, AsPC-1, PANC-1, BxPC-3, CFPAC-1 and SW1990 was estimated by Western blot. 100 ug of cellular proteins were separated on a 10% SDS-PAGE gel, and transferred onto a PVDF membrane. Antibodies specific for GLI1 were used to probe the immunoblots. The blots were then re-probed with β-actin antibody as a loading control. (**B**&**C**) The expression of GLI1 and HIF-1α at mRNA level was evaluated by qRT-PCR. The expression of each target gene was quantified using GAPDH as a normalization control. Results are representative of three independent experiments. Column: mean; bar: SD.

To explore the possible roles of Hh pathway and HIF-1α in the triggering of EMT progress in pancreatic cancer cell lines. We first explored the expression of GLI1 and HIF-1α in six human pancreatic cancer cell lines. As shown in Figure 1A, all pancreatic cancer cell lines except SW1990 express readily detectable levels of GLI1 protein, similar results of the GLI1 mRNA levels in these cell lines were detected using qRT-PCR (Figure 1B). Furthermore, the expression of HIF-1α was also detectable but differed among the six cell lines analyzed by qRT-PCR (Figure 1C).

### Hypoxia accumulates HIF-1α and potentiates Hh signaling in PANC-1 and BxPC-3 cells

**Figure 2:**
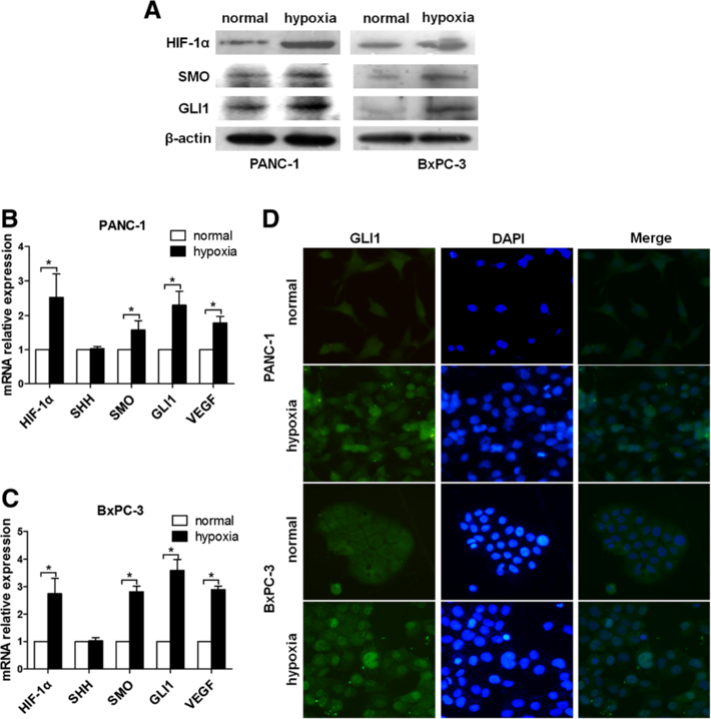
**Effects of hypoxia on HIF-1α and Hh signaling in pancreatic cancer cells.** PANC-1 and BxPC-3 cells were incubated under 3% O_2_ for 48 h prior to harvest. Normal culture was used as negative control. (**A**) Whole cell protein extracts were subject to Western blot analysis using HIF-1α, SMO or GLI1 antibodies. β-actin was used as an internal loading control. (**B**&**C**) Total RNA was extracted and the expression of HIF-1a, SHH, SMO, GIL1 and VEGF were measured by qRT-PCR. The expression of each target gene was quantified using GAPDH as a normalization control. The data represent the results from three independent experiments. (**D**) Immuofluorescence staining of GLI1 in PANC-1, BxPC-3 cells under normoxic or hypoxic conditions for 48 h. Green represents GLI1 staining. Blue signal represents nuclear DNA staining by DAPI. Column: mean; bar: SD; *P < 0.05 compared to normal controls.

Previous studies have shown that the effect induced by hypoxia is mainly mediated by HIF-1α [11]. In order to investigate the effect of hypoxia, 65–70% sub-confluent pancreatic cancer cells (PANC-1, BxPC-3) were exposed to hypoxic conditions (3% O_2_) up to 48 h. As shown in Figure 2A, the expression levels of HIF-1α, SMO and GLI1 proteins were dramatically increased in both two cell lines, compared with normal controls. Moreover, HIF-1α mRNA level dramatically accumulated, and SMO and GLI1 mRNA levels were also significantly elevated in PANC-1 and BxPC-3 cells. However, the level of sonic hedgehog homolog (SHH) mRNA remained unchanged, compared to normal controls (Figure 2B &2C). These results indicated that Hh signaling was activated in both cell lines under hypoxia condition. Additionally, the nuclear translocation of GLI1 was enhanced as an effect of hypoxic exposure, as demonstrated by immunofluorescence (Figure 2D).

### Hypoxia induces an EMT phenotype and promotes invasiveness in pancreatic cancer cells

**Figure 3:**
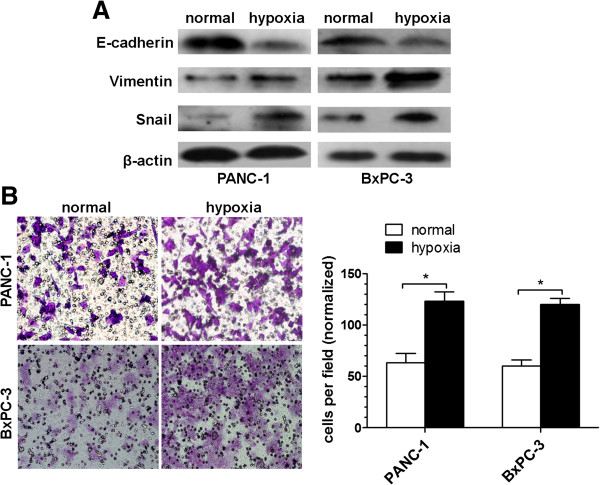
**Characteristics of EMT and invasion under hypoxia condition in pancreatic cancer cells.** (**A**) Western blot analysis of EMT related molecules E-cadherin, vimentin, and Snail of PANC-1, BxPC-3 cells under normoxic or hypoxic conditions for 48 h. (**B**) Matrigel invasion assay. PANC-1 and BxPC-3 cells were seeded into a matrigel-coated invasion chamber under normoxic or hypoxic conditions for 48 h. Left, Representative staining. ×100 magnification. Right, quantification of invasion. The number of migrated cells was quantified by counting the cells from 10 random fields. The data are representative of 3 independent experiments. *P < 0.05. Column: mean (n = 10); bar: SD; *P < 0.05 compared to normal controls.

To investigate whether pancreatic cancer cells underwent EMT as a result of exposure to hypoxia, we examined the expression of markers of epithelial and mesenchymal phenotypes by Western blot. As shown in Figure 3A, hypoxia cells displayed decreased E-cadherin level and increased vimentin and Snail levels. Cancer cells that have undergone EMT tend to exhibit greater invasiveness. On the basis of this premise, we investigated the invasion ability of both normoxic and hypoxic cells by Matrigel invasion assay. Hypoxia exposure significantly increased pancreatic cancer invasion (Figure 3B).

### Silencing of HIF-1α reverses the effects of hypoxia on Hh signaling, EMT process and invasion in pancreatic cancer cells

**Figure 4:**
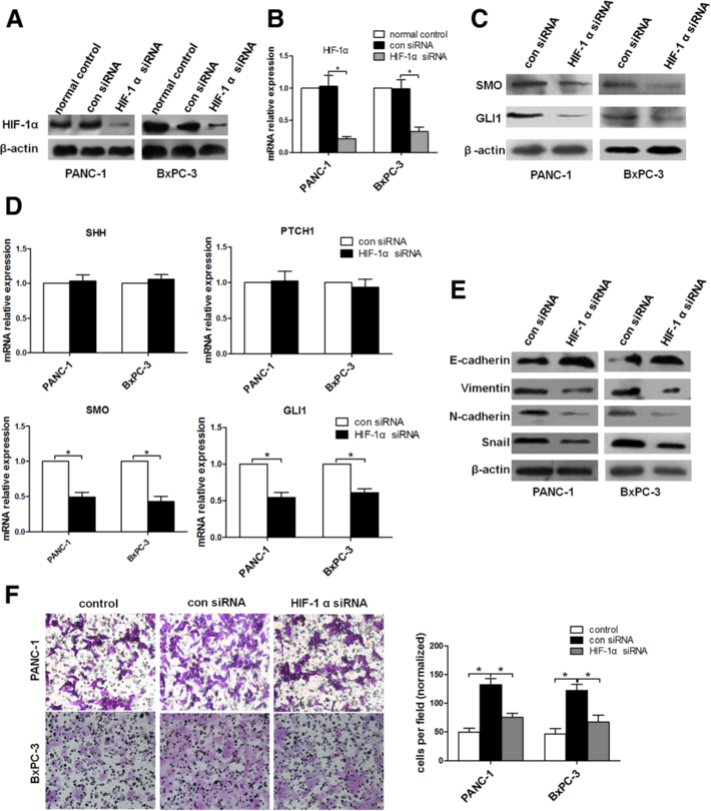
**HIF-1α interference reverses hypoxia induced Hh signaling activation, EMT process and invasion.** PANC-1 and BxPC-3 cells were incubated under hypoxic conditions. Normal control groups were also under hypoxic conditions. (**A**) Western blot detection of HIF-1α in siRNA-transfected PANC-1 and BxPC-3 cells. (**B**) qRT-PCR analysis of HIF-1α mRNA level after both cells transfected with siRNA. (**C**) The effects of HIF-1α siRNA on the expression of SMO and GLI1. SMO and GLI1 expression levels following transfected with siRNA for 48 h were estimated by Western blot. (**D**) The expression of SHH, PTCH1, SMO and GLI1 mRNA level were evaluated by qRT-PCR following transfection with siRNA for 48 h. (**E**) The effects of HIF-1α siRNA on EMT process. E-cadherin, vimentin, N-cadherin and Snail protein levels of PANC-1, BxPC-3 cells transfected with siRNA for 48 h were analysed by Western blot. (**F**) The effect on cell invasion in response to HIF-1α knockdown. After transfection with siRNA for 48 h, PANC-1 and BxPC-3 cells were incubated under hypoxic conditions for 48 h. Control groups were under normoxic condiions. The number of cells was counted under a light microscope.

In order to further investigate the role of HIF-1α in the effects induced by hypoxia, we transiently silenced HIF-1α in the cell lines in hypoxic conditions (Figure 4A-B). Prominent decrease in HIF-1α expression significantly down-regulated the expression levels of SMO and GLI1 in both PANC-1 and BxPC-3 cells, whereas no effect was observed in the expression of SHH and PTCH1 (Figure 4C-D). These data indicate that activated Hh signaling under hypoxia exposure is inhibited by silencing of HIF-1α.

We further delineated the link between hypoxia induced HIF-1α expression and EMT progress. Silencing of HIF-1α resulted in marked decrease in the expression of N-cadherin, vimentin and Snail, but a significant increase in the expression of E-cadherin (Figure 4E), consistent with the reversion to an epithelial phenotype.

To determine the role of HIF-1α in the enhanced invasive capacity of pancreatic cancer cells as a result of exposure to hypoxia, cells were treated with HIF-1α siRNA for 48 h in hypoxia condition prior to the test for invasion. A significantly decreased invasion was observed from HIF-1α silenced hypoxic cells, compared to control cells (Figure 4F). These results demonstrate that the increased invasive ability of cancer cell lines observed in hypoxia was dependent of HIF-1α.

### Hypoxia mediates pancreatic cancer EMT progress and invasion through increasing the expression of SMO

Since hypoxia simultaneously induces tumor cell EMT, invasion and Hh signaling activation without affecting SHH expression, we hypothesized that hypoxia contributes to increased pancreatic cancer cell EMT and invasion through a SMO-dependent manner Hh signaling. To test our hypothesis, pancreatic cancer cells incubated in hypoxia condition were treated with or without either cyclopamine (a SMO antagonist) or GLI1 siRNA to inhibit Hh signaling, and then compared the resulting phenotype with control-treated cells.

**Figure 5:**
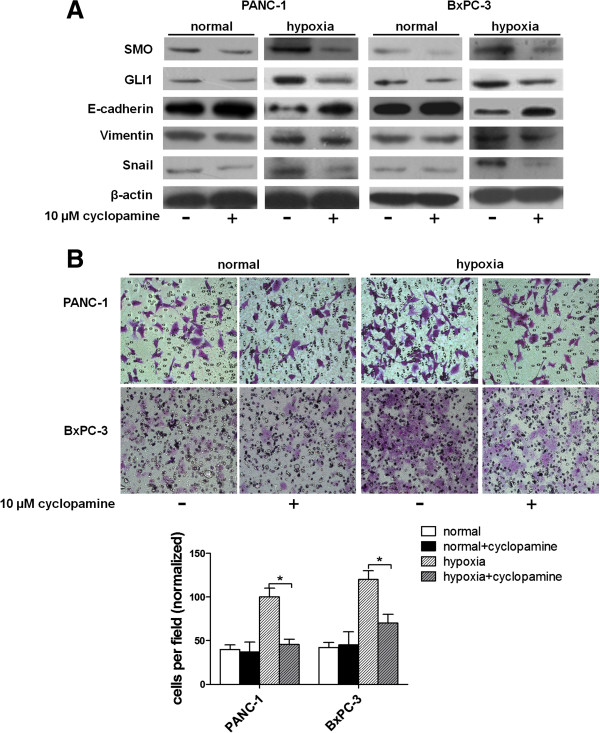
**The effects of cyclopamine on hypoxia induced pancreatic cancer cell EMT and invasion.** PANC-1 and BxPC-3 cells were treated with or without cyclopamine under hypoxia or normal conditions. (**A**) The effects of cyclopamine on the expression of SMO, GLI1 and EMT-related molecules E-cadherin, vimentin and Snial were estimated by Western blot. (**B**) The effect on cell invasion in response to inhibition of SMO by cyclopamine. After treated with cyclopamine for 48 h, the cells were seeded into a matrigel-coated invasion chamber under hypoxia or normal conditions for 48 h. The number of migrated cells was quantified by counting the number of cells from 10 random fields at ×100 magnification.

Under hypoxia exposure conditions, cyclopamine significantly reduced the expression of both SMO and GLI1, and reversed the down-regulation of E-cadherin and up-regulation of Snail. Interestingly, vimentin level was unaffected (Figure 5A). Furthermore, cyclopamine significantly decreased pancreatic cancer invasion induced by hypoxia (Figure 5B). In normal conditions, however, cyclopamine seems have no such significant effect (Figure 5A-B). SMO and GLI1 decreased a little in response to it, and E-cadherin increased slightly. Vimentin, Snail and invasive ability stayed unchanged. These findings suggest that SMO plays a vital role in hypoxia-induced EMT and invasion in pancreatic cancer.

**Figure 6:**
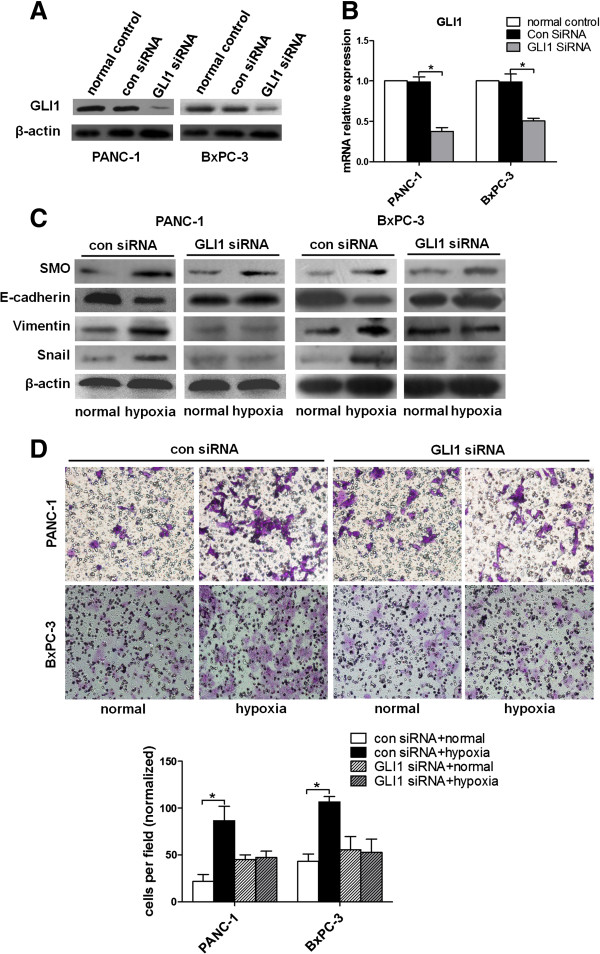
**GLI1 interference abolishes hypoxia induced pancreatic cancer EMT and invasion.** After transfection with siRNA for 48 h, PANC-1 and BxPC-3 cells were incubated under normoxic or hypoxic conditions for 48 h. (**A**) Western blot detection of GLI1 in siRNA-transfected PANC-1 and BxPC-3 cells. (**B**) qRT-PCR analysis of GLI1 mRNA level after both cells transfected with siRNA. (**C**) The effects of GLI1 siRNA on SMO and EMT process. SMO, E-cadherin, vimentin and Snail protein levels of PANC-1, BxPC-3 cells transfected with siRNA were analysed by Western blot. (**D**) The effect on cell invasion in response to GLI1 knockdown. After transfection with siRNA for 48 h, the cells were seeded into a matrigel-coated invasion chamber under normoxic or hypoxic conditions for 48 h. The number of cells was counted under a light microscope.

To further confirm if hypoxia could alter SMO expression earlier than GLI1 in Hh signaling, GLI1 siRNA was applied to knockdown GLI1 in pancreatic cells (Figure 6A-B). And then EMT parameters and invasion were tested. E-cadherin levels were notably increased, while expressions of vimentin and Snail were obviously decreased, even though SMO expression was still up-regulated by hypoxia in GLI1 siRNA groups compared with siRNA control (Figure 6C). Additionally, GLI1 siRNA significantly abolished pancreatic cancer invasion induced by hypoxia (Figure 6D). These results implied that the induced EMT progress and invasion of pancreatic cancer in the presence of hypoxia was significantly abolished in the condition of GLI1 knockdown. Since the blockade of GLI1 does not affect SMO expression, these data indicate that hypoxia facilitates pancreatic cancer cell EMT and invasion through increasing the transcription level of SMO.

## Discussion

Epithelial to mesenchymal transition is described as a dynamic and reversible biological process. In recent years, it has become increasingly clear that EMT plays important roles in the progression of cancer [32]. Several factors, including hypoxia could induce this phenomenon via mediating snail transcription [14,33]. A hypoxic microenvironment is commonly found in the central region of solid tumors, including pancreatic cancer. The correlation between hypoxia and EMT has been previously reported, and HIF-1a has been found to mediate this phenomenon. However, the molecular mechanisms of how HIF-1a mediates EMT process have been largely undefined, although evidence in support of the ability of HIF-1a to activate Nuclear Factor-kB and Notch signaling to induce EMT process has been recently described in several human epithelial cancer cells [12,34].

Previous study showed that hypoxia could activate canonical Hh signaling through accumulation of HIF-1α *in vitro* and *in vivo*[28,29]. Here, we show that accumulated HIF-1α could also trigger non-canonical Hh signaling to facilitate hypoxia induced EMT and invasion processes. A recent report showed that high expression of VEGF, a HIF-1α target gene, facilitates EMT through promoting Snail nuclear localization in prostate cancer [35]. In this study, our data also show that mRNA level of VEGF was significantly up-regulated by hypoxia in pancreatic cancer cells. Furthermore, we demonstrate that the EMT program attributable to hypoxia is largely driven by activation of the Hh signaling pathway. This EMT program is characterized by vimentin and Snail expression and E-cadherin suppression, a highly invasive and mesenchymal phenotype. A previous study showed that knockdown of GLI1 abrogates characteristics of epithelial differentiation, enhances cell motility, and synergizes with TGF-β to induce EMT progress [36]. Intriguingly, EMT conversion of pancreatic cancer cells occurred without up-regulation of Snail or Slug, two canonical inducers of EMT in many other settings, and GLI1 directly regulates E-cadherin transcription, a vital determinant of epithelial tissue feature [36]. In this study, we show that RNAi-mediated GLI1 interference inhibits the hypoxia-induced EMT and decreases cell invasion. Moreover, Snail expression is dramatically reduced, whereas both E-cadherin mRNA and protein levels are notably increased. This difference might be resulted from the distinct culture conditions used: it is possible that pancreatic cancer cells under hypoxia exposure produce enough cofactors interacting with Hh signaling to mediate the EMT progress and invasion.

The Hh signaling is affiliated with EMT, invasion and metastasis in both non-neoplastic and cancer cells [36-39], probably via directly participating in cell migration and angiogenesis [20]. Recently, it is reported that Hh paracrine signaling is required for epithelial tumor cells conducting signals to the stroma in pancreatic cancer [27,40]. However, under conditions of ligand blocking, how Hh signaling is activated in pancreatic cancer cells themselves is undefined, even though paracrine Hh signaling plays a vital role in triggering tumor-associated stroma relying on a ligand-dependent manner in pancreatic cancer. The results here provide noteworthy evidences that the Hh signaling is potentiated through a ligand-independent manner leading to cancer cell EMT and invasion.

**Figure 7:**
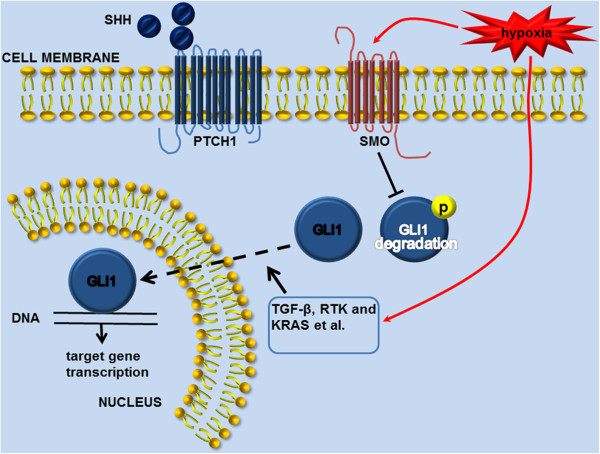
**Schematic diagram of Hh signaling activation under hypoxia condition.** Hypoxia could trigger SMO without affecting SHH or PTCH1 expression. This results in eventual inhibition of factors that promote Gli1 phosphorylation/degradation, and permits cellular accumulation of Gli1. Moreover, hypoxia may trigger other factors, such as TGF-β, KRAS and RTK, to activate GLI1 directly via mechanisms that may operate independently of SMO. Nuclear accumulation of Gli1, in turn, influences transcriptional activity of target genes.

Multiple components of Hh signaling could regulate the pathway at different levels. Cyclopamine could especially bind to SMO heptahelical bundle to inhibit its activity so as to suppress Hh signaling. To determine whether SMO or GLI1 is directly regulated by hypoxia, we exposed pancreatic cancer cells to cyclopamine or GLI1 siRNA in the presence of hypoxia. Although both treatments dramatically reduced tumor invasion and reversed EMT progress induced by hypoxia, GLI1 siRNA could not interrupt the hypoxia-mediated increase in SMO; conversely, blocking SMO function by cyclopamine decreased the expression of the transcription factor GLI1. We also observed that the expression of SHH was not influenced by hypoxia and HIF-1α interference under hypoxia condition also did not affect expression of both SHH and PTCH1. Moreover, a previous report showed that hypoxia could directly elevate SMO expression level to activate Hh signaling, not in a ligand dependent manner [41]. These results indicate that hypoxia activates Hh signaling via up-regulation of SMO expression (Figure 7). Furthermore, GLI1 interference inhibited EMT progress with significantly suppressed vimentin expression, whereas inhibition of SMO through cyclopamine could not reduce vimentin level. These data indicate that hypoxia could, to some extent, bypass SMO to activate GLI1 directly. It is possible that GLI1 transcription is partly decoupled from upstream SHH-PTCH-SMO signaling and is regulated by TGF-β, KRAS and RTK [42-44] (Figure 7). Additionally, nuclear expression of GLI1 was elevated as exposed to hypoxia. These data suggest that it is probably nuclear GLI1 that directly mediate hypoxia-induced EMT and invasion (Figure 7).

Although our data support the hypothesis that Hh signaling pathway is critical for hypoxia-induced EMT and invasion of pancreatic cancer cells, we cannot rule out the possibility that other factors are also involved in hypoxia-induced EMT and invasion. This is because inhibition of SMO by cyclopamine could not reduce vimentin levels, Thus, we speculate that hypoxia enhances EMT and invasion of pancreatic cancer cells through activating a multifaceted factors in which the Hh signaling pathway is a part of an essential network.

## Conclusions

EMT is a key driving force for tumor growth and recurrence. And hypoxia is often experienced by solid tumors, and has been closely linked to EMT and invasion of cancers. Using two pancreatic cancer cell lines, we have demonstrated that non-canonical Hh signaling is required as an important role to switch on hypoxia-induced EMT and invasion in pancreatic cancer cells. Thus, hypoxia mediated-Hh signaling may play an important role in the initiation of EMT and represent a promising therapeutic target for preventing pancreatic cancer progression. Especially, the development of HIF-1α, SMO or GLI1 inhibitor may provide a new class of potent and selectively anticancer agents.

## Methods

### Cell culture and reagents

Pancreatic cancer cell lines were purchased from ATCC (Manassas, VA, USA) and were cultured at 37°C, 5% CO_2_ and 95% air in Dulbecco’s modified Eagle’s medium (DMEM) (high Glucose) (HyClone, Logan, USA) containing 10% heat-inactivated fetal bovine serum (FBS) plus 100 μg/ml ampicillin and 100 μg/ml streptomycin. In experiments designed to assess the role of hypoxia, cells were first cultured in normoxic conditions to obtain the desired subconfluence level (65–70%) and then were incubated in strictly controlled hypoxic conditions (3% O_2_), as previously detailed elsewhere [30,31] for up to 48 h. Cyclopamine, an antagonist of SMO, was obtained from Selleck Chemicals (Houston, USA). Pancreatic cancer cells at exponential phase were cultured in six orifice plates in DMEM supplemented with 1% FBS for 24 h. The drugs (or solvent only) at given concentrations were then added in medium containing 1% FBS, and cells were incubated for another 48 h before a matrigel invasion assay. Antibodies were obtained from the following resources: anti-HIF-1α antibody (Bioworld, Atlanta, GA, USA), anti-SMO antibody (Bioworld), anti-GLI1 antibody (Santa Cruz Biotechnology, Santa Cruz, USA), anti-E-cadherin antibody (Santa Cruz Biotechnology), anti-vimentin antibody (Bioworld), anti-Snail antibody (Santa Cruz Biotechnology), anti-N-cadherin antibody (Santa Cruz Biotechnology), and anti-β-actin antibody (Santa Cruz Biotechnology).

### Cell invasion assay

A chamber based invasion assay (Millipore co., Billerica, USA) was performed to evaluate pancreatic cancer cell invasion. Briefly, the upper surface of the membrane was coated with matrigel (BD Biosciences, Franklin Lakes, USA). Pancreatic cancer cells (1 × 10^5^) were resuspended in upper chamber in serum-free media and allowed to migrate towards a serum gradient (10%) in the lower chamber. The media was aspirated from the inside of the insert and the non-invasive cells on the upper side were removed by scraping with a cotton swab. The membrane was fixed with 4% paraformaldehyde and stained with crystal violet. The number of migrating cells was counted in 10 random fields on each membrane and photographed at ×100 magnification. Values reported here are the averages of triplicate experiments.

### Western blot analysis

Pancreatic cancer cells were washed with ice-cold PBS and were lysed *in situ* with a buffer containing Tris (40 mM, pH 7.4), 10% glycerol, b-glycerophosphate (50 mM), ethylenediaminetetraacetic (5 mM), ethylenediaminetetraa- cetic acid (2 mM), vanadate (0.35 mM), NaF (10 mM), 0.3% Triton X-100, and protease inhibitors (Roche, Penzberg, Germany). After incubation on ice for 30 min, with vortexing every 10 min, cell lysates were centrifuged at 12 000 r.p.m. for 15 min at 4°C. 100 μg of cellular proteins were separated on a 10% SDS-PAGE gel, and the proteins were transferred to the PVDF membranes (Roche). Membranes were blocked with 5% non-fat dry milk in TBST (10 mM Tris-HCl, pH 8.0, 150 mM NaCl, 0.05% Tween 20) and were then incubated with primary antibodies overnight at 4°C. After washing five times for 10 min each in TBST, membranes were incubated with HRP-conjugated secondary antibodies for 2 h, washed again and the peroxidase reaction was performed by an enhanced chemiluminescence detection system to visualize the immunoreactive bands.

### Quantitative real-time PCR assay (qRT-PCR)

Total RNAs were extracted from pancreatic cancer cells using TRIzol reagent (Invitrogen, CA, USA), and the reverse transcription was developed using a PrimeScript RT reagent Kit (TaKaRa, Dalian, China) according to the manufacturer's instruction. The real-time experiments were carried out using the iQ5 Multicolor Real-Time PCR Detection System (Bio-Rad, Hercules, CA) and a SYBR Green PCR Kit (TaKaRa). Following program was used: denaturation at 95°C for 30 sec and 40 cycles consisting of denaturation at 95°C for 5 sec, annealing at 60°C for 30 sec, and extension at 72°C for 30 sec. A melting curve analysis was applied to assess the specificity of the amplified PCR products. The PCR primer sequences for HIF-1α, SHH, PTCH1, SMO, GLI1, E-cadherin, vimentin, Snail, VEGF and GAPDH are shown in Additional file 1: Table S1. The amount of each target gene was quantitated by the comparative C (T) method using GAPDH as the normalization control [45].

### RNA interference

siRNA for HIF-1α (HIF-1α-Homo-2258: 5′-*CCACCACUGAUGAAUUAAATT*-3′, 5′-*UUUAAUUCAUCAGUGGUGGTT*-3′), siRNA for GLI1 (GLI1-Homo-2758: 5′-*GGCUCAGCUUGUGUGUAAUTT*-3′, 5′-*AUUACACACAAGCUGAGCCTT*-3′) and a negative control siRNA (NC: 5′-*UUCUCCGAACGUGUCACGUTT*-3′, 5′-*ACGUGACACGUUCGGAGAATT*-3′) were purchased from GenePharm (Shanghai, China). Cells (2 × 10^5^ per well) seeded in six-well plates were transfected with 100 nM siRNA using Lipofectamine RNAi MAX Reagent (Invitrogen, CA, USA) according to the manufacturer’s instructions. The cells were used for further experiments at 48 h after transfection.

### Immunofluorescence microscopy

After designated treatment, pancreatic cancer cells were fixed with 4% paraformaldehyde for 10 min at room temperature, permeabilized in 0.5% Triton X-100 for 10 min, and blocked in 1% BSA for 1 h. Fixed cells were then incubated with Rabbit anti-human-GLI1 antibodies at 1:100 dilution at 4°C overnight. Cells were washed and incubated with Goat anti-rabbit FITC (green) IgG antibody (ZSGB-BIO Inc., Beijing, China) at 1:100 dilution for 60 min. Nuclei were stained with DAPI for 5 min. The cells were visualized by a fluorescent microscope (Nikon, Japan) using appropriate excitation and emission spectra at ×400 magnification.

### Statistical analysis

Data are presented as the mean ± standard error. Differences were evaluated using one-way ANOVA with the LSD post hoc test for multiple comparisons with SPSS (version 13.0; SPSS, Chicago, IL, USA). P-values below 0.05 were considered statistically significant. In all figures, (*) denotes P < 0.05. All experiments were repeated independently at least three times.

## Competing interest

The authors declare that they have no competing interests.

## Authors’ contributions

JL, XL, HL, QX, WD, and QM designed the experiments. JL, XL, HL, QX, WD, and JM performed the experiments. JL, JM, QM, XL, HL, QX, WD, QS, JX, ZW, and EW analyzed the data. JL, JM, QS, and EW wrote the manuscript. All authors approved the final draft of this manuscript.

## Supplementary Material

Additional file 1: Table S1 Primers for real-time PCR. Click here for file
