# Supplementary material for: Hedgehog signaling regulates hypoxia induced epithelial to mesenchymal transition and invasion in pancreatic cancer cells via a ligand-independent manner
Source: Mol Cancer. 2013 Jun 20;12:66. doi: 10.1186/1476-4598-12-66 (PMC3699387; doi:10.1186/1476-4598-12-66)
Supplement: Additional file 1: Table S1 — Primers for real-time PCR. [file 1476-4598-12-66-S1.doc]

**Supplementary Materials**

Table S1. Primers for real-time PCR

| **genes** | **forword primer** | **reverse primer** |
| --- | --- | --- |
| HIF-1α | 5′-AAGTCTAGGGATGCAGCA -3′ | 5′-CAAGATCACCAGCATCATG-3′ |
| SHH | 5’-TCCAGAAACTCCGAGCGATTTAAG-3’ | 5’-CACTTCCTGGCCACTGGTTCA-3’ |
| PTCH1 | 5′- CCACAGAAGCGCTCCTACA-3′ | 5′- CTGTAATTTCGCCCCTTCC-3′ |
| SMO | 5′-ACGAGGACGTGGAGGGCTG-3′ | 5′-CGCACGGTATCGGTAGTTCT-3′ |
| GLI1 | 5′-GGGATGATCCCACATCCTCAGTC-3′ | 5′-CTGGAGCAGCCCCCCCAGT-3′ |
| E-cadherin | 5′-ATTCTGATTCTGCTGCTCTTG-3′ | 5′-AGTCCTGGTCCTCTTCTCC-3′ |
| vimentin | 5′-AATGACCGCTTCGCCAAC-3′ | 5′-CCGCATCTCCTCCTCGTAG-3′ |
| Snail | 5′-CTTCTCCTCTACTTCAGTCTCTTC-3′ | 5′-CGTGTGGCTTCGGATGTG-3′ |
| VEGF | 5′- ACATCTTCCAGGAGTACCC-3′ | 5′- CTTGGTGAGGTTTGATCCG-3′ |
| GAPDH | 5′-ACCACAGTCCATGCCATCAC-3′ | 5′-TCCACCACCCTGTTGCTGAT-3′ |
